# Adherence Patterns and Health Outcomes in Spanish Young Women Participating in a Virtual-Guided HIIT Program: Insights from the Randomized Controlled WISE Trial

**DOI:** 10.3390/healthcare12191961

**Published:** 2024-10-01

**Authors:** Irene Ferrando-Terradez, Constanza San Martín Valenzuela, Lirios Dueñas, Enrique Alcántara, Alejandro Sendín-Magdalena, Yasmin Ezzatvar

**Affiliations:** 1 Department of Physiotherapy, University of Valencia, 46010 Valencia, Spain; ifete@alumni.uv.es (I.F.-T.); lirios.duenas@uv.es (L.D.); 2 Unit of Personal Autonomy, Dependency and Mental Disorder Assessment, Faculty of Medicine, University of Valencia—INCLIVA Biomedical Research Institute, 46010 Valencia, Spain; 3 Centro Investigación Biomédica en Red de Salud Mental, CIBERSAM, Av. Monforte de Lemos, 3-5, 28029 Madrid, Spain; 4 Physiotherapy in Motion, Multi-Specialty Research Group (PTinMOTION), Department of Physiotherapy, University of Valencia, 46010 Valencia, Spain; 5 Lifestyle Factors with Impact on Ageing and Overall Health (LAH) Research Group, Department of Physiotherapy, University of València, 46010 Valencia, Spain; 6 Data Driven Innovation and Association of the Valencian Community for Driving R&D and Impact Innovation in Sports (4icvesport), 46010 Valencia, Spain; qalcantara@4icvesport.org; 7 Department of Physiotherapy, Faculty of Health Sciences, European University of Valencia, 46010 Valencia, Spain; alejandro.sendin@universidadeuropea.es; 8 Lifestyle Factors with Impact on Ageing and Overall Health (LAH) Research Group, Department of Nursing, University of València, 46010 Valencia, Spain; yasmin.ezzatvar@uv.es

**Keywords:** virtual-guided exercise, high-intensity interval training, young women, physical activity, adherence, quality of life, quality of sleep

## Abstract

Background/Objectives. A notable decline in physical activity from late adolescence to early adulthood affects young women especially. This study aimed to investigate adherence to an unsupervised virtual-guided high-intensity interval training (HIIT) exercise program among young women in Spain. Methods. A total of 106 participants were recruited and randomized to the Women’s Involvement in Steady Exercise (WISE) trial exercise program, administered remotely via a mobile app, and a control group. The primary outcome was adherence measured through daily steps. Secondary variables were patient-reported outcomes of physical activity, quality of sleep, and quality of life, assessed with the International Physical Activity Questionnaire (IPAQ), the Pittsburgh Sleep Quality Index (PSQI), and the Health Lifestyle and Personal Control Questionnaire (HLPCQ), respectively. The assessments were performed over 6 months, before the intervention (T0), at the halfway of the program (T1), and at the end (T2). Results. Daily steps revealed a decrease from baseline to final assessment in both groups. Secondary outcomes indicated a shift in physical activity levels, showing a transition from low to moderate and high activity perception. While sleep quality deteriorated post-intervention, quality of life showed no changes. Conclusions. The WISE trial highlights the potential and challenges of an unsupervised virtual-guided HIIT program for young women. While an improvement in physical activity levels was noticed, it also led to a decrease in daily steps and poorer sleep quality. These findings suggest a complex relationship between exercise and lifestyle factors, which could potentially have the greatest impact.

## 1. Introduction

The benefits of physical activity for holistic health are well documented. However, there is an ongoing public health challenge due to the widespread decline in physical activity, particularly among young adults, leading to increased risks of cardiovascular, metabolic, and musculoskeletal diseases. This is further exacerbated by sedentary lifestyles, which contribute to negative health outcomes. Numerous studies indicate a sharp decrease in physical activity during key life transitions, particularly from late adolescence to early adulthood, with young women experiencing a more pronounced decline compared to men. Gender-specific barriers such as body image concerns, lack of time, and societal pressures have been cited as factors influencing lower levels of physical activity among women [1,2]. Given this, addressing the unique needs and challenges faced by young women is critical for designing effective interventions aimed at improving physical activity adherence and overall health outcomes.

In addition to the physical benefits, physical activity has a significant impact on various aspects of emotional and psychological well being, which translates into improvements in quality of life. Previous studies have documented that exercise programs, including HIIT, can have positive effects on overall well being, as they influence multiple areas such as personal satisfaction, mental health, and social well being [3,4,5]. HIIT interventions have been shown to lead to moderate improvements in mental well being, depression severity, and perceived stress compared to non-active controls [3]. Furthermore, engaging in HIIT produces statistically significant improvements in physical, mental, and overall quality of life in both clinical and non-clinical populations [4]. Research has also demonstrated that HIIT and moderate-intensity training can significantly reduce stress, anxiety, and depression while increasing resilience [5]. Given that quality of life is an integral component of overall well being, this study includes its measurement as a key variable, aiming to assess not only the physical effects of exercise but also its benefits in terms of perceived health and emotional well being. The rationale for measuring sleep quality and self-reported physical activity lies in their role as potential mediators that may influence quality of life.

Adherence to exercise programs is essential for their success. The World Health Organization (WHO) defines adherence as the degree to which a person’s behavior corresponds with healthcare recommendations [6]. In the realm of the exercise literature, adherence is considered successful when participants complete at least two-thirds of the prescribed exercise routine [7]. However, despite well-designed programs, adherence remains a challenge, with many individuals discontinuing their exercise routines relatively quickly [8]. This underscores the need for targeted interventions that consider both the physical and psychological barriers to adherence in specific populations, such as young women.

In this regard, technology has emerged as a promising tool to enhance personalized exercise programs. Mobile health (mHealth) wearables and applications offer virtual guidance, remote support from health professionals, and real-time biometric feedback, such as heart rate monitoring [9]. Studies examining apps designed to improve adherence and health outcomes have shown promising results, particularly in facilitating self-monitoring and providing personalized feedback [10,11]. These tools often incorporate features like reminders, motivational messages, and exercise tracking logs [12,13], which have been linked to improved adherence rates. However, despite these advancements, previous research on unsupervised exercise interventions with virtual guidance has noted high dropout rates [14,15,16]. This suggests that while technology can be an effective tool, more innovative strategies are required to maintain long-term engagement in physical activity.

The type of exercise modality also plays a role in adherence. High-intensity interval training (HIIT) has been shown to offer advantages over other exercise modalities by optimizing the time/benefit ratio, making it particularly appealing for individuals with time constraints [17,18]. Studies have highlighted that HIIT not only provides significant physiological benefits, such as improvements in cardiovascular fitness and metabolic health, but also maintains higher levels of exercise enjoyment compared to other forms of exercise [19]. These attributes suggest that HIIT could be an effective strategy to improve adherence, especially when combined with virtual guidance and remote support.

Despite these insights, there remains a significant gap in the literature regarding the effectiveness of unsupervised, technology-assisted exercise programs in promoting long-term adherence among young women. The lack of evidence on this topic underscores the necessity of further research to explore the potential of such interventions in improving not only adherence but also broader health outcomes, such as quality of life and sleep quality. Without this knowledge, it is challenging to design interventions that address the specific needs of this population.

 **Hypothesis 1.**  *We hypothesize that an unsupervised HIIT intervention with virtual guidance will improve exercise adherence, physical activity levels, and quality of life among young women compared to a control group, despite potential challenges in maintaining sleep quality.*

For the above reasons, this study aims to analyze the effects of an unsupervised HIIT intervention with virtual guidance on adherence among young women. A secondary objective is to evaluate the impact of the intervention on self-reported behavior related to physical activity, sleep quality, and quality of life and compare the results with a control group.

## 2. Materials and Methods

### 2.1. Trial Design

The Women’s Involvement in Steady Exercise (WISE) protocol [20], about an unsupervised exercise intervention with virtual guidance for young physically inactive women, was followed in this work. The WISE trial is a randomized controlled study conducted across three centers, designed as a single-blind trial with an intervention period lasting six months (NCT05467280) and an allocation ratio of 1:1. This paper presents the results based on data collected at the Spanish study center. All procedures were approved by the Ethics Committee of the University of Valencia (No. 1944476) in accordance with the World Medical Association’s Declaration of Helsinki, ensuring the ethical conduct of the research. Once ethics approval was obtained, the trial was conducted in Valencia, Spain. The experimental intervention was administered remotely through a mobile app, allowing participants to complete the exercise program independently. However, all assessments were conducted face to face at the Physiotherapy Department of the University of Valencia. Written informed consent was obtained from all participants prior to the commencement of this study. Recruitment and data collection occurred from August 2022 to February 2023. No modifications were made to the original protocol intervention or recruitment criteria once this study commenced. The CONSORT guidelines were followed in preparing this manuscript, despite the repeated measures design (Supplementary Material Table S1).

### 2.2. Participants

Eligible participants were young women who met the following inclusion criteria [20]: (1) age between 15 and 24 years, (2) sedentary, defined by non-compliance with WHO physical activity recommendations [21], and a score < 1 on the International Physical Activity Questionnaire (IPAQ) [22]. Exclusion criteria included [20] (1) a diagnosis of diabetes, (2) cardiac problems incompatible with exercise, (3) unwillingness to wear the smartwatch during the six-month intervention, and (4) a history of severe COVID-19 [23]. Participants were recruited from local schools and universities via email and posters, with the assistance of local authorities.

### 2.3. Randomization, Allocation, and Blinding

Participants were assigned to one of two groups based on a computer-generated randomization list, with an equal 1:1 allocation ratio. The group assignments were communicated using sealed opaque envelopes, labeled as either control (0) or intervention (1). Randomization was conducted by an external technician who was not affiliated with this study and who also was responsible for sending exercise videos, educational advice, and motivational activities notifications through the app to the intervention group. Assessors were blinded to the intervention status, as variables were derived from objective data provided by the smartwatch and patient-reported outcome measures (PROMs). The researcher who performed the data analysis was also blinded to the participant’s randomization.

### 2.4. Intervention

Participants in the intervention group followed the WISE trial exercise program [20] over a 6-month period, which was delivered remotely through a mobile app specifically developed for this study (Google Play: WISE project v1.0.10: https://play.google.com/store/apps/details?id=com.kineticanalysis.wise&hl=es_419&pli=1, accessed on 22 September 2024). Each exercise session, guided by a sports science professional, lasted between 20 and 30 min and adhered to a structured format: 5–7 min for warm-up, 10–15 min of high-intensity interval training (HIIT), and 5–7 min for cool-down stretching. Starting from the tenth session, exercises were offered at two different intensity levels, allowing participants to select the one that matched their fitness level. The intensity of the exercises was gradually increased with each session to maintain variety. Alongside the virtual exercise sessions, participants received weekly educational content through the WISE app, which included information on nutrition, sleep, and overall well being. To boost motivation, social media groups and weekly live streaming sessions were organized, covering topics such as nutrition and physical activity and addressing any concerns related to the WISE app workouts. Additionally, participants in the intervention group utilized the Xiao Mi Band 5 smartwatch, which was synchronized with the WISE app to monitor their exercise progress, track their weight, and record their sleep patterns.

In contrast, the control group was provided with general physical activity guidelines, initial instructions [20], and access to the activity monitoring tool offered through the smartwatch. Furthermore, at the end of this study, they were given access to exercise videos and educational messages.

### 2.5. Outcomes

To assess adherence, the primary outcome measured was the daily step count over a six-month period, as tracked by the Xiaomi Mi Band 5 smartwatch [24,25]. The Xiaomi Mi Band 5 has demonstrated adequate validity for step measurement during low-intensity exercise and high reliability for monitoring physical activity, particularly among low-cost trackers [24,25]. Daily step counts were assessed at three time points: (1) the baseline (T0_steps_), representing the mean number of steps recorded over the 15 days prior to the start of the WISE program; (2) the first three months (T1_steps_), representing the mean number of steps recorded during the first three months of the WISE program; and (3) the last three months (T2_steps_), representing the mean number of steps recorded during the final three months of the intervention. Days with incomplete data or those recording fewer than 1000 steps were omitted from the analysis to maintain the accuracy and reliability of the data collected. Adherence was additionally assessed using four metrics recorded in a weekly online exercise diary. (1) Completion is the number of participants who consistently followed the exercise videos and attended all scheduled assessments; (2) attendance is the percentage of videos completed by participants; (3) duration is a binary measure indicating whether participants engaged in at least 20 min of exercise twice a week; and (4) intensity is the average perceived exertion reported by participants after each session, measured using the modified Borg scale.

As secondary variables, patient-reported outcome measures (PROMs) were assessed before the start of the intervention (T0), at the halfway mark of the program (T1), and at the end of the program after six months of intervention (T2). Self-reported daily life physical activity (outside of the WISE program) was evaluated using the IPAQ, which assesses walking and moderate-to-vigorous activities performed continuously for at least 10 min over the past 7 days [26]. The IPAQ categorizes activity into low, moderate, and high, with good test–retest reliability and fair-to-moderate correlation with accelerometer data [27].

In addition, quality of life was measured using the Health Lifestyle and Personal Control Questionnaire (HLPCQ). The HLPCQ comprises 26 items scored on a 4-point Likert scale, with a total score ranging from 26 to 104 points. Higher scores indicate a healthier lifestyle [28]. In terms of reliability, the internal consistency of the Polish version and its domains is excellent. Cronbach’s alpha for each of the domains of the scale ranged between 0.6 and 0.9 [29]. Lastly, quality of sleep was assessed using the Pittsburgh Sleep Quality Index (PSQI), a widely used questionnaire consisting of 19 items that collectively form a global score ranging from 0 to 21 [30]. Higher scores indicate poorer sleep quality, while lower scores suggest better sleep quality. In terms of psychometric properties, the Spanish version of the PSQI used with non-professional caregivers demonstrated an internal consistency of 0.75, indicating acceptable reliability [31]. Additionally, a validation study in an adolescent population reported a Cronbach’s alpha of 0.73, with moderate-to-large positive correlations between the PSQI global score and the CES-D (r = 0.58) and SCAS (r = 0.45) total scores, supporting its construct validity [32]. In Table 1, we can see a detailed overview of the schedule for participant enrollment, interventions, and assessments of all the outcomes throughout this study.

### 2.6. Sample Size

Sample size was estimated in order to attend the primary aims of this publication and was based on anticipated changes in daily step count (small effect size, f = 0.16) following physical activity interventions and smartphone applications [33]. G*Power software v3.1.9.6 [34] was used, and a type I error rate of 5% and a power of 90% were considered, resulting in a required sample size of 84 participants. Considering a 20% dropout rate, the initial recruitment target was set at 101 participants.

### 2.7. Data Analyses and Statistical Methods

Statistical analyses were conducted using SPSS version 24 (SPSS Inc., Chicago, IL, USA). Continuous variables were presented as means and standard deviations if they fulfilled the normality assumption. In contrast, categorical outcomes were expressed as percentages. Demographic variables (age, weight, height, and body mass index) were described and analyzed using *t*-tests to compare participants who completed the WISE program and control group.

To assess changes in the primary outcome (daily steps) and quality of life and quality of sleep secondary outcomes (through the HLPCQ and PSQI questionnaires), a two mixed-factor Multivariate Analysis of Variance (MANOVA) was employed, with time serving as the within-subject factor (T0, T1, T2) and group (intervention versus control) as the between-subject factor. Before performing the MANOVA analysis, the assumptions of normality, sphericity, homoscedasticity, and equality of multiple variance–covariance matrices were explored through the Kolmogorov–Smirnov/Kruskal–Wallis, Mauchly, Levene, and Box tests, respectively. Bonferroni corrections were applied for post hoc comparisons. The threshold for statistical significance was established at *p* < 0.05, with exact *p*-values and 95% confidence intervals provided.

For the IPAQ questionnaire, a non-parametric Friedman test was used due to categorical responses, and a Wilcoxon test was used for pairwise comparisons when the time factor effect was analyzed. On the other hand, to explore the group effect on the IPAQ questionnaire, the Mann–Whitney U test was employed in each assessment time. The statistical significance of pairwise comparisons of nonparametric tests was adjusted according to the number of comparisons performed.

## 3. Results

**Figure 1:**
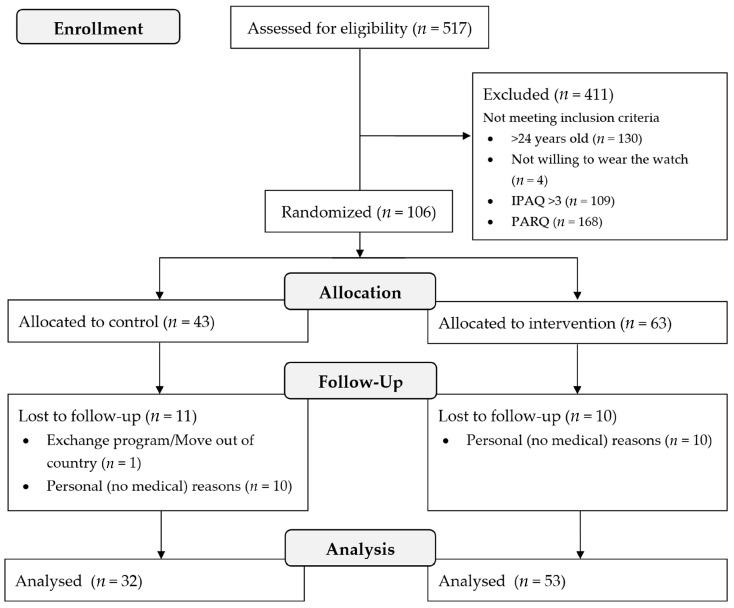
CONSORT flow diagram illustrating the recruitment, follow-up of participants across this study, and final numbers of people included in the analysis.

Initially, 106 women were recruited who started the WISE trial. However, 21 participants dropped out this study (Figure 1). Table 2 provides a description of the sample and data on adherence to the exercise program for those who completed this study. Among the women in the intervention group, 53.67% completed all the videos from the WISE program, while 35.3% completed at least 20 min of every video. The perceived exertion, measured by the modified Borg scale, was 6.34.

The interaction of the factors time and group was not statistically significant (*p* > 0.05) for daily steps, the HLPCQ, and the PSQI outcomes. On the other hand, for the primary and secondary variables, the time factor was statistically significant for daily steps (F_(1.61; 133.92)_ = 15.43; *p* < 0.01; η^2^_p_ = 0.16) and the PSQI (F_(1.84; 152.73)_ = 24.65; *p* < 0.01; η^2^_p_ = 0.23) but not for the HLPCQ (*p* = 0.89). Table 3 shows the participant’s performance and the differences between time. Both groups showed a significant decrease in the number of steps at the end of this study (*p* < 0.05). In the same way, both groups perceived a statistically significant worse quality of sleep at times T1 and T2 (*p* < 0.05). The group factor did not have a significant effect on daily steps (*p* = 0.12), the PSQI (*p* = 0.07), or the HLPCQ (*p* = 0.71).

In relation to physical activity outside the WISE program reported with the IPAQ questionnaire, both the intervention group (χ^2^_(2)_ = 46.97; *p* < 0.01) and the control group (χ^2^_(2)_ = 16.28; *p* < 0.01) presented an effect due to the time factor. As Table 3 shows, the perception of exercise was significantly higher during the program period than before starting the WISE program, reaching a high perception of the level of physical activity in 50.94% of the intervention group. Although the control group reaches a high level of physical activity in 38.71% and 43.75% of the sample at the midterm and post-intervention, respectively, the groups do not differ statistically at any of the measurement times.

## 4. Discussion

The primary objective of the WISE trial was to investigate adherence to an unsupervised virtual-guided HIIT exercise program among a cohort of young women in Spain. The results revealed a decrease in daily step counts from the baseline to the final assessment and between the midterm and final evaluation. This decrease contrasts with the findings from previous research that indicated interventions utilizing step count monitoring devices typically lead to increases in daily step counts over both short- and long-term periods [35]. Several factors might account for these discrepancies, such as the potential for overtraining, which could cause fatigue and a subsequent decline in daily physical activity [36]. At the same time, being engaged in training, regardless of its intensity, may have led the participants to experience sufficient satisfaction that did not create an additional need to maintain a high level of physical activity for the rest of the day. However, the control group experienced the same pattern of daily steps; hence, the variable weather conditions added to the academic demands of the time in which the intervention was developed (from August 2022 to February 2023) could be a major factor in the outcome. The program’s start in September coincided with favorable weather in Valencia, Spain, and participants returning from summer vacations were likely well rested. However, the later months included colder weather and periods of academic exams, which could explain the observed reduction in daily steps and sleep quality in both groups. This may indicate the need to strengthen the education of young participants regarding continued physical exercise beyond motivation, including teaching theoretical knowledge about the effects of exercise at a technical level.

In terms of measurement methods, this study utilized the Xiaomi Mi Band 5 smartwatch to assess daily step counts, which proved to be a reliable tool for tracking physical activity. While more commonly used brands include Fitbit, Garmin, and Apple, the Xiaomi Mi Band 5 has been validated for its accuracy in measuring steps and heart rate across various activities [37,38,39]. This choice was based on the device’s demonstrated reliability and affordability, making it a practical option for large-scale studies. In addition, this study’s use of an online exercise platform aligns with the growing evidence supporting the effectiveness of virtual interventions in promoting behavior change and improving physical health [40,41,42,43,44]. It is essential to acknowledge that measurement tools and methods can influence adherence and outcomes. Our study focused on measuring adherence through duration, attendance, and intensity, aligning with recommendations from Hawley-Hague et al. [7] to effectively monitor and mitigate dropout rates. Future studies should consider incorporating a range of devices and approaches to enhance the robustness of adherence measurements and ensure comprehensive evaluations of exercise interventions.

Regarding daily life physical activity levels, outside the WISE sessions training, the IPAQ questionnaire revealed a shift from low to moderate and high physical activity levels following the exercise program. Even when groups showed no differences in the IPAQ questionnaire through time, at the midterm, the intervention’s participants reported a greater tendency to experience a high level of physical exercise. Nonetheless, it should be noted that the IPAQ’s comprehensive nature in assessing several daily activities could likely contribute to this observed increase [26]. It is noteworthy that this increase might be influenced by other areas of the participants’ daily routines beyond their engagement with the WISE program itself. This suggests that the program may have indirectly influenced broader patterns of physical activity rather than the number of daily steps only; therefore, future studies should consider this type of variables.

On the other hand, the results from the HLPCQ showed no significant changes in quality of life post-intervention, which is consistent with findings from Wang et al. [45]. This lack of change may indicate that the HLPCQ is not sufficiently sensitive to detect subtle improvements in quality of life resulting from the exercise intervention. Future research might explore alternative or more refined measures for evaluating quality of life in the context of physical activity programs to capture these potential nuances. On the contrary, the sleep quality measured through the PSQI results indicated a deterioration in sleep quality post-intervention, as evidenced by increased scores. This outcome can be explained by the potential rise in core body temperature following intense exercise, which has been associated with reduced sleep efficiency and increased wakefulness [46]. Additionally, factors such as seasonal changes, colder weather, and academic pressures during the intervention period may have contributed to poorer sleep quality. While some research suggests that regular exercise can improve sleep quality and duration [47,48], our findings were contrary. This discrepancy may be due to the bidirectional nature of the relationship between exercise and sleep, where poor sleep might also contribute to decreased physical activity levels [49]. Optimizing exercise timing and the type of exercise could potentially enhance the positive effects of physical activity on sleep quality.

Regarding exercise adherence, this study found that 80.19% of the intervention group followed the exercise videos and attended all assessments. Among those who did not drop out, 53.67% completed all the videos, and 35.3% exercised for at least 20 min twice a week. The mean perceived exertion, measured using the modified Borg scale, was 6.34. This moderate level of exertion may have influenced participants’ motivation, potentially reducing the perceived benefits of the exercise program. Higher exertion levels might lead to more noticeable fitness improvements, thereby enhancing motivation and adherence.

Finally, this study acknowledges several limitations. Firstly, the analysis did not account for potential confounding factors, such as holiday seasons or temperature variations, that could impact adherence. Secondly, the menstrual cycle was not controlled for, despite its potential influence on physiological and psychological responses to exercise, such as perceived exertion, fatigue, or mood. Future studies could consider exploring this variable to better understand its impact on exercise adherence and related outcomes, particularly in young women. Thirdly, this study did not assess the long-term effects of the intervention, which is crucial for understanding the sustained impact of the exercise program. Future research should address these limitations by considering these external factors, controlling for menstrual cycle phases, and extending the evaluation period to provide a more comprehensive understanding of how to maintain engagement and optimize outcomes in exercise programs for young women.

## 5. Conclusions

The WISE trial highlights the potential and challenges of an unsupervised virtual-guided HIIT program for young women in Spain. While the intervention improved moderate physical activity levels, it also led to a decrease in daily step counts and poorer sleep quality. These findings suggest a complex relationship between exercise and lifestyle factors, emphasizing the need for a nuanced understanding of how virtual interventions affect participants’ overall well being.

To our knowledge, this is the first study to use a virtual HIIT program for young women remotely without direct supervision and to report on the physical activity adherence not only to the training but also to the daily life of the participants. This is especially relevant for the strategies of future studies to facilitate the transfer of interventions’ results into the daily life of the study population, as our unexpected results point out the complex relationship between exercise and lifestyle factors. Therefore, future physical exercise interventions should include also the intervention of the sample’s habits, considering contextual variables, and exploring long-term effects to enhance the effectiveness and sustainability of such interventions. Additionally, investigating the role of social support and motivational factors could further inform strategies to improve adherence and outcomes. Despite its limitations, this study contributes valuable insights into virtual exercise programs and their role in supporting health behavior change, ultimately paving the way for more tailored and effective approaches for promoting physical activity among young women.

## Figures and Tables

**Table 1:** Schedule of enrolment, interventions, and assessments.

	STUDY PERIOD							
	**Selection Visit**	**T0**	**Intervention**					
TIMEPOINT	*M-*_1_	0	*M*_1_	*M*_2_	*M*_3_/*T*1	*M*_4_	*M*_5_	*M*_6_/T2
ENROLMENT:								
Eligibility screen	X							
Informed consent	X							
Randomization		X						
INTERVENTIONS:								
Intervention group			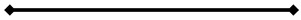					
Control group			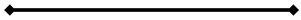					
ASSESSMENTS:								
Step count			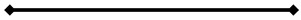					
Adherence			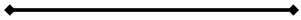					
IPAQ		X			X			X
HLPCQ		X			X			X
PSQI		X			X			X

X, represents the specific time points when assessments or key study activities are carried out during the study period; 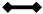, show the duration and timeline for when specific interventions or assesments are applied throughout the study period, the diamond shapes mark the start or the end of the period; IPAQ, International Physical Activity Questionnaire; HLPCQ, Healthy Lifestyle and Personal Control Questionnaire; PSQI, Pittsburgh Sleep Quality Index.

**Table 2:** Description of the sample and adherence to the WISE program for women who completed the training.

	Intervention		Control		Group (No Drop Out) Differences (*p*-Value)
	No Drop Out	Drop Out	No Drop Out	Drop Out	
**Anthropometric outcomes**					
Age (years)	21.10 (1.95)	20.50 (2.36)	21.04 (1.74)	21.44 (1.50)	0.95
Weight (kg)	62.60 (10.65)	60.06 (11.10)	56.81 (6.95)	63.25 (13.20)	0.21
Height (m)	1.61 (0.06)	1.61 (0.05)	1.63 (0.07)	1.58 (0.05)	0.12
BMI	23.50 (3.76)	23.14 (5.05)	21.35 (2.74)	24.97 (4.72)	0.01 *
**Adherence description to the virtual-guided HIIT program**					
Completion (%)			80.19		
Duration (%)			Yes: 35.3; No: 64.7		
Attendance (%)			53.67 (20.22); Me: 54		
Intensity (modified Borg scale score)			6.34 (1.41); Me: 6.42		

Values are expressed as mean and standard deviation [M (SD)]. Adherence description also includes the median (Me). Categorical outcomes are expressed with percentage frequency. BMI, body mass index. Completion: % of participants who followed the exercise videos and attended all assessments. Duration: % of participants that perform a minimum of 20 min of exercise 2 times/week. Attendance: % of videos completed. * Indicates differences between the intervention and control group, considering the participants who did not drop out.

**Table 3:** Dependent outcomes registered during this study.

Outcomes	Intervention Group			Control Group		
	T0	T1	T2	T0	T1	T2
Daily steps (steps/day)	8665.83 (3089.58) **	7757.46 (1992.95) **	7128.80 (2029.34)	7676.66 (2167.79) **	7275.00 (1859.27) **	6718.03 (1612.63)
HLPCQ (total score/104)	61.06 (9.42)	63.14 (8.66)	61.70 (12.34)	64 (9.61)	61.13 (8.46)	61.97 (10.50)
PSQI (total score/21)	6.62 (3.26)	8.72 (2.66) *	8.64 (2.65) *	5.73 (2.69)	7.70 (2.50) *	7.47 (2.83) *
IPAQ (level)	Low: 49.06%	Low: 5.66% *^,^**	Low: 20.00% *	Low: 46.88%	Low: 16.13% *	Low: 28.13% *
	Mod.: 50.94%	Mod.: 43.40%	Mod.: 42.0%	Mod.: 53.13%	Mod.: 45.16%	Mod.: 28.13%
	High: 0%	High: 50.94%	High: 38.0%	High: 0%	High: 38.71%	High: 43.75%

Data are expressed as mean (SD, standard deviation). IPAQ, International Physical Activity Questionnaire. Mod., moderate. HLPCQ, Health Lifestyle and Personal Control Questionnaire. PSQI, Pittsburgh Sleep Quality Index. Statistical significance was set at *p* < 0.05. * Indicates statistically significant differences with T0. ** Indicates statistically significant differences with T2.

## Data Availability

The original contributions presented in the study are included in the article/Supplementary Material, further inquiries can be directed to the corresponding author/s.

## Supplementary Materials

The following supporting information can be downloaded at https://www.mdpi.com/article/10.3390/healthcare12191961/s1, Table S1: CONSORT checklist.
