# Supplementary material for: Adherence Patterns and Health Outcomes in Spanish Young Women Participating in a Virtual-Guided HIIT Program: Insights from the Randomized Controlled WISE Trial
Source: Healthcare (Basel). 2024 Oct 1;12(19):1961. doi: 10.3390/healthcare12191961 (PMC11475678; doi:10.3390/healthcare12191961)
Supplement: Supplementary file 1 [file healthcare-12-01961-s001.zip › healthcare-3212176-supplementary.pdf]

CONSORT 2010 checklist of information to include when reporting a randomised trial

Adherence Patterns and Health Outcomes in Spanish Young Women Participating in a Virtual-Guided HIIT Program: In-sights from the Randomized Controlled WISE Trial

Section/Topic	Item Nº	Checklist item	Reported on
**Title and abstract**			
	1a	Identification as a randomised trial in the title	Line 4
	1b	Structured summary of trial design, methods, results, and conclusions	Lines 24-41
**Introduction**			
Background and objectives	2a	Scientific background and explanation of rationale	Lines 46-108
	2b	Specific objectives or hypotheses	Lines 109-111
**Methods**			
Trial design	3a	Description of trial design (such as parallel, factorial) including allocation ratio	Lines 119-136
	3b	Important changes to methods after trial commencement (such as eligibility criteria), with reasons	-
Participants	4a	Eligibility criteria for participants	Lines 138-145
	4b	Settings and locations where the data were collected	Lines 129-131
Interventions	5	The interventions for each group with sufficient details to allow replication, including how and when they were actually administered	Lines 158-177
Outcomes	6a	Completely defined pre-specified primary and secondary outcome measures, including how and when they were assessed	Lines 179-219
	6b	Any changes to trial outcomes after the trial commenced, with reasons	-
Sample size	7a	How sample size was determined	Lines 227-232
	7b	When applicable, explanation of any interim analyses and stopping guidelines	-
Randomisation:			
Sequence generation	8a	Method used to generate the random allocation sequence	Lines 147-152
	8b	Type of randomisation; details of any restriction (such as blocking and block size)	Lines 147-152
Allocation concealment mechanism	9	Mechanism used to implement the random allocation sequence (such as sequentially numbered containers), describing any steps taken to conceal the sequence until interventions were assigned	Lines 147-152
Implementation	10	Who generated the random allocation sequence, who enrolled participants, and who assigned participants to interventions	Lines 147-152

Blinding	11a	If done, who was blinded after assignment to interventions (for example, participants, care providers, those assessing outcomes) and how	Lines 152-157
	11b	If relevant, description of the similarity of interventions	-
Statistical methods	12a	Statistical methods used to compare groups for primary and secondary outcomes	Lines 234-255
	12b	Methods for additional analyses, such as subgroup analyses and adjusted analyses	-
**Results**			
Participant flow (a diagram is strongly recommended)	13a	For each group, the numbers of participants who were randomly assigned, received intended treatment, and were analysed for the primary outcome	Figure 1, Line 263
	13b	For each group, losses and exclusions after randomisation, together with reasons	Figure 1, Line 263
Recruitment	14a	Dates defining the periods of recruitment and follow-up	Lines 132-133
	14b	Why the trial ended or was stopped	-
Baseline data	15	A table showing baseline demographic and clinical characteristics for each group	Table 2, Lines 267-273
Numbers analysed	16	For each group, number of participants (denominator) included in each analysis and whether the analysis was by original assigned groups	Figure 1, Line 263
Outcomes and estimation	17a	For each primary and secondary outcome, results for each group, and the estimated effect size and its precision (such as 95% confidence interval)	Table 3, Lines 291-295
	17b	For binary outcomes, presentation of both absolute and relative effect sizes is recommended	Table 3
Ancillary analyses	18	Results of any other analyses performed, including subgroup analyses and adjusted analyses, distinguishing pre-specified from exploratory	-
Harms	19	All important harms or unintended effects in each group	-
**Discussion**			
Limitations	20	Trial limitations, addressing sources of potential bias, imprecision, and, if relevant, multiplicity of analyses	Lines 368-379
Generalisability	21	Generalisability (external validity, applicability) of the trial findings	-
Interpretation	22	Interpretation consistent with results, balancing benefits and harms, and considering other relevant evidence	Lines 297-367
**Other information**			
Registration	23	Registration number and name of trial registry	Lines 412
Protocol	24	Where the full trial protocol can be accessed:	https://clinicaltrials.gov/study/NCT05467280?term=NCT05467280&rank=1
Funding	25	Sources of funding and other support (such as supply of drugs), role of funders	Lines 408-409
